# Mid-Trimester Maternal Serum hCG and Alpha Fetal Protein Levels: Clinical Significance and Prediction of Adverse Pregnancy Outcome

**DOI:** 10.5812/ijem.5014

**Published:** 2013-04-01

**Authors:** Georgios Androutsopoulos, Panagiotis Gkogkos, Georgios Decavalas

**Affiliations:** 1 Department of Obstetrics and Gynaecology, University of Patras, Medical School, Rion, Greece

**Keywords:** Maternal Serum Screening Tests, Chorionic Gonadotropin, AFP, Adverse Pregnancy Outcome

## Abstract

**Context:**

Maternal serum human Chorionic Gonadotropin (hCG) and Alpha Fetal Protein (AFP) were originally introduced to detect trisomy 21 and neural tube defects. However, in the absence of aneuploidy or neural tube defects, mid-trimester maternal serum hCG and/or maternal serum AFP associated with adverse pregnancy outcomes. Pregnancies with unexplained mid-trimester elevation in maternal serum hCG and/or maternal serum AFP, are at increased risk for pregnancy complications resulting from placental insufficiency.

**Evidence Acquisition:**

Mid-trimester maternal serum hCG>2.5 MoM associated with an increased risk for pregnancy complications including: late fetal loss, gestational hypertension, preeclampsia, intrauterine growth restriction (IUGR), preterm delivery and intrauterine fetal death(IUFD). Mid-trimester maternal serum AFP levels >2.5 MoM are thought to reflect a defect in placentation and associated with an increased risk for pregnancy complications including: late fetal loss, gestational hypertension, preeclampsia, IUGR, preterm delivery and IUFD.

**Results:**

Combined mid-trimester elevation in maternal serum hCG and AFP levels suggest a more complex type of placental pathology. They have stronger association with pregnancy complications including: late fetal loss, gestational hypertension, preeclampsia, IUGR, preterm delivery and IUFD.

**Conclusions:**

Mid-trimester maternal serum hCG or AFP levels alone cannot detect all pregnant women with increased risk to develop pregnancy complications. Multiparameter testing of placental function in mid-trimester (maternal serum hCG and AFP screening, uterine artery Doppler and placental morphology) may allow us to identify women with increased risk to develop severe placental insufficiency and pregnancy complications. However, future prospective studies are needed to confirm the prognostic significance of multiparameter testing of placental function in mid-trimester.

## 1. Context

Maternal serum human Chorionic Gonadotropin (hCG) and Alpha Fetal Protein (AFP) were originally introduced to detect trisomy 21 and neural tube defects. However, increased ultrasound machine quality and sonographer expertise, has greatly reduced the need for maternal serum hCG (ms-hCG) and maternal serum AFP (ms-AFP) screening in mid-trimester (1, 2).

In the absence of aneuploidy or neural tube defects, mid-trimester ms-hCG and/or ms-AFP associated with adverse pregnancy outcomes (3). Pregnancies with unexplained mid-trimester elevation in ms-hCG and/or ms-AFP, are at increased risk for pregnancy complications resulting from placental insufficiency [intrauterine growth restriction (IUGR), preeclampsia, intrauterine fetal death (IUFD)] (4).

### 1.1. Maternal Serum hCG

hCG is a member of the glycoprotein hormone family [hCG, Follicle Stimulating Hormone (FSH), Luteinizing Hormone (LH), Thyroid Stimulating Hormone (TSH)] (5). All of them are dimers consisting of a common α-subunit and distinct β-subunits that are associated noncovalently (5-7). The distinct β-subunits confer biological activity and display various degrees of homology (5). In adults hCG expression is often associated with pregnancy (6). However, hCG can be found in other conditions such as gestational trophoblastic disease and non-germinomatous germ cell tumors (6, 8). During pregnancy hCG is produced almost exclusively by the syncytiotrophoblast of the placenta (6). However, it is synthesized by the fetal kidney and fetal liver (9). Most of the hCG in circulation is metabolized by the liver (10). Also, 20% of the circulating hCG is excreted by the kidneys (10).However, hCG appears early during pregnancy (6). Its concentration increases gradually by reaching a peak at 8 to 10 weeks of gestation (3, 4,6). After that peak, it progressively declines to reach a plateau at 18 to 20 weeks of gestation (3, 4,6). During early pregnancy, the main function of hCG is the maintenance of the corpus luteum (3, 4). It takes over from LH in promoting progesterone production by ovarian corpus luteal cells, preventing menstrual bleeding (7). It promotes progesterone production only for 3-4 weeks following pregnancy implantation (7). Also hCG receptor gene expressed by uterine spiral arteries and hCG acts on them promoting angiogenesis of uterine vasculature and uterine growth in line with fetal growth (7). During pregnancy hCG also promotes: cytotrophoblast differentiation, immunosuppression and blockage of phagocytosis of invading trophoblast cells (7, 11,12).

### 1.2. Maternal Serum AFP

AFP is a glycoprotein and it is a member of the albuminoid gene family [AFP, albumin (ALB), alpha albumin (αALB), vitamin D binding protein (DBP)] (13, 14). In adults AFP expression is often associated with hepatocellular cancer, non-germinomatous germ cell tumors and gastrointestinal cancers (13). However, AFP can be found in non-neoplastic conditions such as hepatitis, cirrhosis and pregnancy (13, 14). During pregnancy it is synthesized by the fetus (13, 14). AFP is normally produced in early pregnancy primarily by the fetal liver and yolk sac (15, 16). It is also produced to a lesser extent by the fetal gastrointestinal tract (15, 16). As the yolk sac involutes at the 9th week of gestation, the fetal liver is the principal source of AFP during development (15, 16). AFP synthesis by the proliferating fetal liver actually increases through the 20th week of gestation, after which it remains fairly constant until 32nd week (15-17).AFP is excreted into fetal urine and transported to maternal serum through the placenta or by diffusion across fetal membranes (14, 18). The transplacental passage of AFP involves additional and more complicated mechanisms (14). It is asymmetrical and unidirectional, displaying a faster transfer rate of AFP from the fetal to maternal circulation (14). Small amounts of AFP is transported and can be measured in the maternal serum (13). Despite the decrease in fetal serum AFP (fs-AFP) during mid-trimester of gestation, ms-AFP continues to rise until the 32nd week of gestation (15-17). After the 32nd week of gestation, ms-AFP begins to decline until parturition (17). AFP is involved with ontogenic and oncogenic growth (13, 14). It can bind and transport a multitude of ligands (bilirubin, fatty acids, retinoids, steroids, heavy metals, dyes, flavonoids, phytoestrogens, dioxin and various drugs) (14). Also, it is capable of regulating growth in reproductive, hematopoietic, placental, hepatic, inflammatory and lymphatic cells (14). However, AFP regulating function during pregnancy remains controversial and relatively unknown (19). AFP involvement in the regulation of placental growth, is also unknown.

## 2. Evidence Acquisition

### 2.1. Mid-Trimester Elevation of Maternal Serum hCG

Elevated mid-trimester ms-hCG levels have been associated with congenital abnormalities, placental dysfunction and adverse pregnancy outcome (3). Although there are various cut-off points, the most common cut-off values are between 2 and 2.5 MoM(20). However there are no significant differences among them (21, 22). In the absence of fetal chromosomal or structural anomalies, mid-trimester ms-hCG > 2.5 MoM associated with an increased risk for pregnancy complications including: late fetal loss [OR 2.2 (95% CI: 1.3-3.0)], gestational hypertension [OR 1.4 (95% CI: 1.1-1.8)], preeclampsia [OR 1.19 (95% CI: 0.88–1.61)], IUGR [OR 1.3 (95% CI: 0.9-1.7)], preterm delivery [OR 1.7 (95% CI: 1.4-2.1)] and IUFD [OR 2.7 (95% CI: 1.8-4.0)](3, 4, 23-27). The risk of adverse pregnancy outcome increases as the mid-trimester ms-hCG levels become more elevated (3, 23,27). Extremely high mid-trimester ms-hCG levels (≥ 10 MoM) imply a poor pregnancy outcome (27). Mechanisms for elevated mid-trimester ms-hCG levels with a structurally normal fetus include: placental ischemia and inadequate trophoblastic remodeling (26, 28-30). Trophoblastic cells cultured in vitro showed that hypoxia increases hCG production (31). Placental pathology studies suggest that unexplained mid-trimester elevation of ms-hCG levels associated with villitis, placental vascular lesions (infarction, ischemic changes, intervillus thrombosis), velamentous cord insertion and placental mosaicism (26, 28-30, 32, 33). It is unknown whether elevated ms-hCG levels, are the cause or the result of villitis and placental vascular lesions.

### 2.2. Mid-Trimester Reduction of Maternal Serum hCG

Isolated mid-trimester ms-hCG levels < 0.5 MoM have no association with adverse pregnancy outcome (3, 34).

### 2.3. Mid-Trimester Elevation of Maternal Serum AFP

Elevated mid-trimester ms-AFP levels have been strongly associated with congenital abnormalities, placental dysfunction and adverse pregnancy outcome (3, 17). Although there are various cut-off points, the most common cut-off values are between 2 and 2.5 MoM (20). However there are no significant differences among them (21, 35). In the absence of fetal chromosomal or structural anomalies, mid-trimester ms-AFP levels >2.5 MoM are thought to reflect a defect in placentation [placental abruption, placenta previa, abnormal placental adherence (placenta accreta, increta and percreta)] (3, 36, 37). They also associated with an increased risk for pregnancy complications including: late fetal loss [OR 10.1 (95% CI: 7.5-13.5)], gestational hypertension [OR 1.6 (95% CI: 1.3-2.1)], preeclampsia [OR 0.83 (95% CI: 0.44–1.56)], IUGR [OR 2.3 (95% CI: 1.8-2.9)], preterm delivery [OR 1.8 (95% CI: 1.5-2.3)] and IUFD [OR 5.3 (95% CI: 3.8-7.3)] (3, 4, 17, 23-25, 38, 39). Mechanisms for elevated mid-trimester ms-AFP levels with a structurally normal fetus include: disruption of the fetal-maternal placental barrier, placental vascular damage from early abruption, fetal-maternal bleeding and fetal-placental ischemia (3, 36, 40-42).Placental pathology studies suggest that unexplained mid-trimester elevation of ms-AFP levels associated with chorionic villitis and placental vascular lesions (43). It is unknown whether elevated ms-AFP levels, are the cause or the result of these lesions. Probably the factor that leads to adverse pregnancy outcome also increases ms-AFP (3, 38).

### 2.4. Mid-Trimester Reduction of Maternal Serum AFP

Mid-trimester ms-AFP levels < 0.25 MoM have been associated with late fetal loss [OR 15.1 (95% CI: 9.3-24.8)], preterm delivery [OR 2.2 (95% CI: 1.3-3.8)], stillbirth [OR 4.0 (95% CI: 1.0-16.0)] and macrosomia (3, 23, 38, 44, 45).

2.5. Mid-Trimester Combined Elevation of Maternal Serum hCG and AFP

Although isolated abnormal mid-trimester ms-hCG or ms-AFP levels associated with an increased risk for with congenital abnormalities and adverse pregnancy outcome, the risk increases when they are combined (3, 4, 24, 25, 46). The risk for adverse pregnancy outcome also increases as mid-trimester ms-hCG and/or ms-AFP levels become more extreme. In the absence of fetal chromosomal or structural anomalies, combined mid-trimester elevation in ms-hCG and ms-AFP levels suggest a more complex type of placental pathology which have stronger association with pregnancy complications including: late fetal loss [OR 7.05 (95% CI: 1.18-29.88)], gestational hypertension [OR 0.78 (95% CI: 0.13-3.24)], preeclampsia [OR 6.67 (95% CI: 3.84-11.58)], IUGR [OR 3.54 (95% CI: 1.26-9.14)], preterm delivery [OR 3.20 (95% CI: 1.49-6.61)] and IUFD [OR 6.87 (95% CI: 1.71-22.93)] (3, 4, 23-25, 39, 46, 47). Placental pathology studies suggest that unexplained combined mid-trimester elevation in ms-hCG and ms-AFP levels associated with villitis and placental vascular lesions (48).

## 3. Results

Mid-trimester ms-hCG and/or ms-AFP levels alone cannot be detected in all pregnant women with increased risk to develop pregnancy complications (21, 35,49). Although many of the associations between mid-trimester ms-hCG and/or ms-AFP levels and adverse pregnancy outcomes are statistically significant, the sensitivity and positive predictive value are too low for them to be clinically useful as screening tests (3). All these women with unexplained elevation of mid-trimester ms-hCG and/or ms-AFP levels should receive, increased fetal surveillance (3). There are no proven protocols to manage these women with increased risk to develop pregnancy complications(3, 23,25). The optimal type and frequency of testing, remains undetermined (3, 4, 23,25). Ultrasonography is the most cost-effective approach and should be used as the initial diagnostic examination (3, 4). Blood flow in the uterine arteries during mid-trimester can be evaluated noninvasively using Doppler ultrasonography (3, 50). Uterine artery Doppler measurements show that impedance to blood flow in the uterine arteries progressively decreases with gestational age in normal pregnancies (50, 51). Increased impedance and reduced blood flow in the uterine arteries associated with subsequent development of pregnancy complications characterized by spiral artery vasculopathy (preeclampsia, IUGR, IUFD) (3, 51,52).Also, reduced uteroplacental blood flow is more prevalent in women with elevated mid-trimester ms-hCG and/or ms-AFP levels and may be a useful marker for the subset of women with increased risk to develop pregnancy complications (late fetal loss, gestational hypertension, preeclampsia, IUGR, preterm delivery and IUFD) (3, 23, 48, 53, 54). Likewise, abnormalities of placental shape or texture in women with elevated mid-trimester ms-AFP levels have been associated with poor pregnancy outcome (48). However, uterine artery Doppler screening alone is superior to mid-trimester ms-hCG and ms-AFP screening to identify a significant placental pathology leading to pregnancy complications (55).

## 4. Conclusions

Multiparameter testing of placental function in mid-trimester (ms-hCG and ms-AFP screening, uterine artery Doppler and placental morphology) may allow us to identify women with increased risk to develop severe placental insufficiency and pregnancy complications (4, 21, 35, 48, 49, 56). However, future prospective studies are needed to confirm the prognostic significance of multiparameter testing of placental function in mid-trimester (21, 49,57).
